# An *in vitro* Reconstructed Human Skin Equivalent Model to Study the Role of Skin Integration Around Percutaneous Devices Against Bacterial Infection

**DOI:** 10.3389/fmicb.2020.00670

**Published:** 2020-05-14

**Authors:** Eleonore C. L. Bolle, Anthony D. Verderosa, Rabeb Dhouib, Tony J. Parker, John F. Fraser, Tim R. Dargaville, Makrina Totsika

**Affiliations:** ^1^Tissue Repair and Translational Physiology Program, Institute of Health and Biomedical Innovation, Queensland University of Technology, Brisbane, QLD, Australia; ^2^The Innovative Cardiovascular Engineering and Technology Laboratory, Critical Care Research Group, The Prince Charles Hospital, Brisbane, QLD, Australia; ^3^Infection and Immunity Research Program, Institute of Health and Biomedical Innovation, School of Biomedical Sciences, Faculty of Health, Queensland University of Technology, Brisbane, QLD, Australia

**Keywords:** *Staphylococcus aureus*, 3D skin models, tissue engineered skin, melt electrowriting, percutaneous implant, driveline infections

## Abstract

Percutaneous devices are a key technology in clinical practice, used to connect internal organs to external medical devices. Examples include prosthesis, catheters and electrical drivelines. Percutaneous devices breach the skin’s natural barrier and create an entry point for pathogens, making device infections a widespread problem. Modification of the percutaneous implant surface to increase skin integration with the aim to reduce subsequent infection is attracting a great deal of attention. While novel surfaces have been tested in various *in vitro* models used to study skin integration around percutaneous devices, no skin model has been reported, for the study of bacterial infection around percutaneous devices. Here, we report the establishment of an *in vitro* human skin equivalent model for driveline infections caused by *Staphylococcus aureus*, the most common cause of driveline-related infections. Three types of mock drivelines manufactured using melt electrowriting (smooth or porous un-seeded and porous pre-seeded with human fibroblasts) were implanted in human skin constructs and challenged with *S. aureus.* Our results show a high and stable load of *S. aureus* in association with the skin surface and no signs of *S. aureus*-induced tissue damage. Furthermore, our results demonstrate that bacterial migration along the driveline surface occurs in micro-gaps caused by insufficient skin integration between the driveline and the surrounding skin consistent with clinical reports from explanted patient drivelines. Thus, the human skin-driveline infection model presented here provides a clinically-relevant and versatile experimental platform for testing novel device surfaces and infection therapeutics.

## Introduction

Percutaneous devices are routinely used in medicine to connect internal organs to external medical devices or their components. Examples include drivelines for the transmission of electrical currents to ventricular assist devices (VADs), urinary peritoneal and vascular catheters for the transfer of fluids, or bone protheses for the transfer of forces (von Recum, 1984). VADs are a type of mechanical circulatory support used in heart failure therapy. They are attached to the heart and assist the heart in generating sufficient output to ensure adequate end organ perfusion (Koval and Stosor, 2019). VADs are powered and controlled through an external battery pack and controller unit. The connection between the pump and the battery pack and controller unit is maintained via a driveline, which contains insulated wires to carry current and telemetric data (Feldmann et al., 2018). In the United States alone, a minimum of 2,500 patients are added to the patient registry for mechanical circulatory support in any given year (Kirklin et al., 2017). Percutaneous devices, such as drivelines, cross the skin through a surgical incision and disrupt the skin’s natural barrier creating an entry point for pathogens (von Recum, 1984). Thus, infections around percutaneous devices are highly prevalent with recent studies reporting catheter infection rates to be in the range of 2–33% over the life of the device, depending on the type of catheter (Salwiczek et al., 2014).

Driveline infections, in particular, account for 83% of ventricular assist device-specific infections, with incidence rates of 1.31 and 1.42 driveline infections per 100 patient months at 3 month pre- and post-device implantation, respectively (INTERMACS, 2015). More importantly, driveline infections are associated with an impaired clinical outcome, with patient survival rates only 76% by 24 month after the first driveline infection (Hannan et al., 2019). Driveline infections are primarily caused by bacteria, with fungal infections also reported less frequently (Hannan et al., 2019; Koval and Stosor, 2019). Gram-positive bacteria from the *Staphylococcus* genus are the most common causative agents, with *Staphylococcus aureus* accounting for 28–44% of all driveline infections (Koval and Stosor, 2019).

The most common drivelines currently in clinical practice are made from a smooth material (silicone or polyurethane, depending on the manufacturer) and surrounded by a porous poly(ethylene terephthalate) sleeve, commercialized as Dacron^TM^. The purpose of the Dacron^TM^ sleeve is to increase tissue integration and prevent pathogen entry. While manufacturer guidelines recommend that the Dacron^TM^ sleeve should be implanted so that it interfaces with the skin at the exit site, recent studies have demonstrated significantly reduced infection rates with the Dacron^TM^ implanted subcutaneously and the smooth section of the driveline crossing the skin instead (Singh et al., 2012; Dean et al., 2015; McCandless et al., 2015; Camboni et al., 2016).

Despite clinical findings demonstrating reduced infection rates when smooth driveline sections cross the skin, there is evidence that smooth surfaces do not allow for sufficient skin integration, thus leading to the formation of a sinus tract around the implant (Winter, 1974; von Recum and Park, 1981; Isackson et al., 2011). A sinus tract is formed by the epidermal layer migrating downwards parallel to the device, in an attempt to restore epidermal continuity (von Recum, 1984). The epidermal downwards migration can be reduced by increasing skin integration into the implant through the inclusion of pores to the surface of the percutaneous implant (Winter, 1974; Fukano et al., 2010). The prevailing opinion in the field of percutaneous devices is that increased skin and tissue integration will in turn create a tighter biological seal at the device exist site against invading pathogens and thus decrease infection rates (von Recum, 1984; Tagusari et al., 1998; Choi et al., 1999; Fukano et al., 2010; Isackson et al., 2011).

We have previously shown that porous scaffolds manufactured by melt electrowriting (MEW), an additive manufacturing technique, supports fibroblasts growth (Farrugia et al., 2013) and increases skin integration compared to smooth tubes in a 3D reconstructed human skin equivalent (HSE) model. Moreover, pre-seeding the porous scaffolds with human dermal fibroblasts prior to device implantation in HSEs appeared to reduce epidermal downgrowth, compared to un-seeded porous implants (Bolle et al., 2019). Based on these findings, the aim of the present study was to investigate whether increased skin integration around MEW porous implants can create a better biological seal against pathogen entry. Using a reconstructed HSE model, this is the first study to investigate *S. aureus* driveline-specific infections and provides a versatile *in vitro* platform for the detailed investigation of factors contributing to this significant clinical challenge worldwide.

## Materials and Methods

### Keratinocyte and Fibroblast Cell Isolation and Culture

Keratinocytes were isolated from human skin samples obtained from patients undergoing abdominoplasty surgery and breast reduction surgery (Xie et al., 2010) and cultured according to previously published protocols (Rheinwald and Green, 1975; Dawson et al., 2006; Haridas et al., 2016). In brief, following an enzyme digestion overnight in 0.125% trypsin (Invitrogen, Australia) the epidermal layer was separated from the dermal layer. Keratinocytes were isolated from the epidermal layer and cultured on a layer of irradiated 3T3 fibroblasts in keratinocyte growth medium (Invitrogen, Australia). Fibroblasts were isolated from the dermis and cultured in fibroblasts growth medium, as previously described (Haridas et al., 2016).

### 3D Reconstructed Human Skin Equivalent Preparation and Culture

**FIGURE 1:**
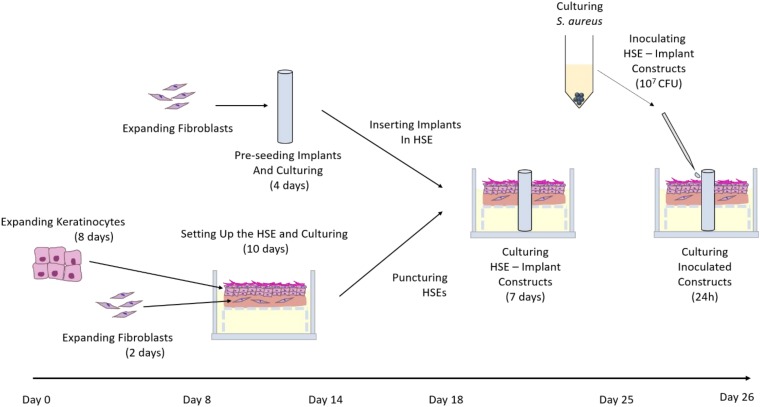
Summary of *in vitro* HSE-driveline infection model establishment. Human primary fibroblasts and keratinocytes were expanded and seeded onto a de-epidermised and de-cellularised dermis. Simultaneously, fibroblasts were expanded, seeded onto drivelines and cultured for 4 days. Skin constructs were punctured on the 10th day of culture and the three types of mock drivelines (smooth PCL, porous un-seeded, and porous pre-seeded) were inserted. The skin-driveline constructs were cultured for another 7 days prior to challenge with 10^7^ CFU directly *S. aureus* ATCC 29213 directly at the skin-driveline interface. At 24 hpi samples were either processed for enumeration of viable bacteria or fixed, sucrose infiltrated and snap frozen for subsequent histological analysis.

The de-epidermised dermis (DED) was prepared following protocols described by Chakrabarty et al. (1999) and Dawson et al. (2006). The DEDs were between approximately 15 × 15 mm and 20 × 20 mm in size and were stored at 4°C in antibiotic/antimycotic medium consisting of DMEM supplemented with 1% v/v penicillin/streptomycin (Invitrogen). Skin constructs, referred to hereafter as HSEs, were generated as described previously (McGovern et al., 2013; Bolle et al., 2019). Briefly, 3.6 × 10^4^ keratinocytes and 1.8 × 10^4^ fibroblasts were seeded onto the DEDs through a stainless-steel ring with an inner diameter of 9 mm and lifted at the air liquid interface 48 h post seeding (Figure 1). HSEs were cultured in keratinocyte growth medium with fresh medium replaced every 48 h. DEDs and cells were not patient-matched.

### Driveline Manufacture

All mock (wireless, i.e., do not carry electrical charge) drivelines utilized in this study were manufactured from medical grade polymer poly(ε-caprolactone; PCL, Corbion Purac, Netherlands). PCL scaffolds were manufactured via MEW as previously described (Bolle et al., 2019) using an in-house custom built machine previously described in Wunner et al. (2017). Briefly, the PCL was loaded into a plastic syringe (Nordson EFD, United States), heated (84°C) and extruded by applying air pressure. A voltage of 6.7 kV was applied to the needle and the jet was collected on a grounded and motorized *x*–*y* collector plate in a lattice with layers at 90° and 45° to each other. The scaffolds were cut into 10 × 10 mm squares using a scalpel.

**FIGURE 2:**
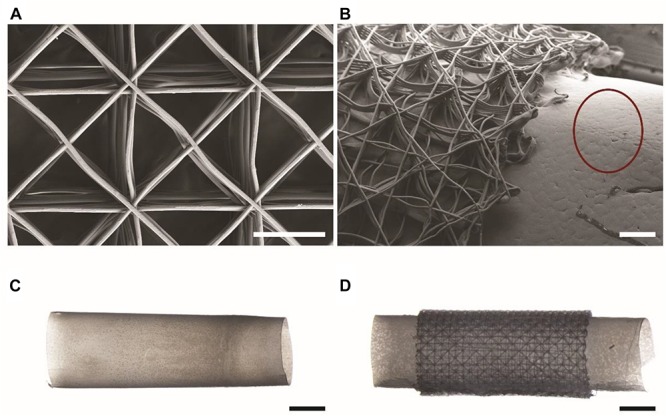
Mock drivelines manufactured by melt electrowriting (MEW). SEM images of **(A)** a MEW scaffold used for constructing porous mock drivelines and **(B)** a porous mock driveline showing the MEW scaffold fused to the PCL tube (scale bars: 300 μm). The red circle highlights the surface morphology of the smooth PCL tube. Stereomicroscope images of **(C)** a smooth PCL tube and **(D)** a porous driveline (MEW scaffold – PCL tube) construct. Scale bars: 2 mm.

To produce mock drivelines with different surfaces, a 4 mm stainless steel mandrel was dip-coated into a 10% w/v solution of PCL in chloroform, as previously described (Bolle et al., 2019). The mandrel was then immersed in ethanol to aid in the removal of the PCL from the mandrel, resulting in smooth, hollow PCL tubes, from now onward referred to as PCL drivelines. To obtain tubular scaffolds, the scaffolds were wrapped around the heated, PCL coated mandrels to fuse the bottom layers of the scaffolds to the PCL core. The PCL tube-scaffold constructs were removed from the mandrel with the aid of ethanol. This resulted in hollow tubes with a solid core and porous outer surface with an outer diameter of 4.5 mm, referred to as un-seeded drivelines from now onward. All drivelines were sterilized by immersion in 80% ethanol for 30 min, dried in a laminar flow hood and exposed to UV light for 20 min on either side. The MEW scaffolds and the PCL tube – scaffold constructs (porous mock drivelines) are shown in Figure 2. Scanning electron microscopy (SEM) images were acquired using a Zeiss Sigma Field Emission SEM, equipped with a Zeiss Gemini column at an accelerating voltage of 5 kV. All samples were gold sputter coated prior to imaging using a Leica EM-SCD005. Stereomicroscope images of the samples were acquired on a Nikon SMZ25 stereomicroscope (Nikon, Japan).

### Pre-seeding of Porous Mock Drivelines With Human Fibroblasts

The tubular scaffolds were seeded with fibroblasts as previously described (Bolle et al., 2019). Briefly, the scaffolds were placed in 12-well plates following an overnight incubation in fibroblasts growth medium and seeded with human dermal fibroblasts isolated according to the above described protocol, by applying 30 μL containing 2 × 10^5^ cells onto the scaffold. Following 30 min of incubation to allow for initial cell attachment, the scaffolds were inverted and another 30 μL containing 2 × 10^5^ cells were placed on the scaffolds. Following a further 30 min incubation the samples were submerged in fibroblasts growth medium and cultured for 4 days, with medium changed every 48 h. The pre-seeded scaffolds are hereafter referred to as pre-seeded drivelines.

### Implanting Human Skin Equivalents With Mock Drivelines

The mock drivelines were inserted into HSEs 10 day post seeding of fibroblasts and keratinocytes, as previously described (Bolle et al., 2019). Briefly, 4 mm holes were created in the HSEs with a biopsy punch (Stiefel) and the mock drivelines inserted from the dermal side and carefully pulled through using forceps (Figure 1). Skin-driveline constructs were cultured at the air liquid interface with medium replaced every 48 h.

### Bacterial Strains and Culture Conditions

*Staphylococcus aureus* ATCC 29213 was used in this study and routinely cultured in Lysogeny broth (LB) medium with shaking (200 RPM) at 37°C. For HSE inoculations, bacterial cultures were collected at 16 h, centrifuged and resuspended in antibiotic-free keratinocyte growth medium at a cell density of 5 × 10^8^ colony forming units (CFU)/mL. An inoculum volume of 20 μL (1 × 10^7^ CFU) was used in all HSE infection assays.

### *S. aureus* Infection Assay of Human Skin Equivalents Implanted With Mock Drivelines

Human skin equivalent culture medium was replaced with antibiotic-free keratinocyte growth medium 72 h prior to infection. Intact HSEs controls (without a driveline) were inoculated by depositing 1 × 10^7^ CFU *S. aureus* ATCC 29213 in a small 20 μL volume at the centre of the construct surface. HSEs with implants were infected by depositing 4 × 5 μL of *S. aureus* inoculum in a cross-shape around the tubular implant interface. The cross-shaped pattern was chosen to ensure that the inoculum volume was evenly distributed around the entire circumference of the mock drivelines. Inoculated HSEs were incubated in antibiotic-free keratinocyte growth medium at 5% CO_2_ and 37°C. At 24 h post inoculation (hpi), HSEs were processed for histology or for viable CFU counts as described in sections below. Time-matched uninfected HSEs served as controls. A schematic overview of the entire experimental timeline is shown in Figure 1.

### Enumeration of Viable *S. aureus* From Infected Human Skin Equivalents

Human skin equivalents collected at 24 hpi were dissected (roughly 3 × 3 mm pieces) under aseptic conditions, weighed and digested in a 1 mg/mL collagenase solution in phosphate buffered saline (PBS; Gibco, Australia) at 37°C for 3 h in a shaking incubator (200 RPM). The samples were then homogenized mechanically in a mini-Beadbeater (Daintree Scientific, Australia) with stainless steel beads (0.5 mm, Daintree Scientific). Samples were homogenized for 15 min in 2 min cycles at 2,500 RPM, with 1 min rest on ice between cycles. HSE homogenates were then serially diluted 10-fold in PBS and viable bacteria (CFU) in samples were enumerated by plating triplicate 5 μL aliquots of each dilution onto LB agar followed by overnight incubation at 37°C.

### Histological Examination of *S. aureus* Infected Human Skin Equivalents

Intact HSEs and HSEs with mock drivelines collected at 24 hpi were prepared for histological analysis as described in Bolle et al. (2019). Briefly, the HSEs were rinsed in PBS supplemented with magnesium and calcium and fixed in 4% PFA for a minimum of 30 min. HSEs were then infiltrated in a sucrose–OCT solution (optimal cutting temperature, Tissue Tek, Finland) with increasing ratios of OCT to preserve tissue morphology. HSEs were then snap frozen in a dry ice-ethanol slush and stored at −80°C until required for further processing. Frozen HSE samples were sectioned at 20 μm thickness on a cryostat (Leica Biosystems) and stained with hematoxylin and eosin (Bolle et al., 2019). Coverslips were applied using prolong gold mounting medium (Life Technologies) and slides were imaged using a Zeiss Axio Imager M2 microscope (Zeiss) or a 3D Histech Scan II Brightfield Slide Scanner (3D Histech, Hungary). For localizing *S. aureus* within the tissue, HSE sections were stained following a published Gram staining protocol, with modifications (Becerra et al., 2016). Briefly, the sections were rinsed, stained with crystal violet for 1 min, incubated with iodine for 2 min, decolorised in acetone and counterstained with safranin for 1 min (ProSciTech, Australia) with a wash performed between each step. Slides were imaged on a Leica DM2500 microscope (Leica, Germany).

### Fluorescence Microscopy of Explanted Mock Drivelines and *S. aureus* Staining

Drivelines were carefully removed from HSEs using forceps immediately after collection at 24 hpi and prepared for imaging of *S. aureus* colonization. Live/dead staining of *S. aureus* was performed using the LIVE/DEAD^TM^ BacLight^TM^ Bacterial Viability kit L7007 (Life Technologies, Australia). Explanted drivelines were rinsed in saline (0.9%) for 30 s, then incubated in the dark at 30°C for 25 min, with SYTO9 and Propidium Iodide (PI; 200 μL). The final applied concentration of SYTO9 and PI was 3.35 μM and 20 μM, respectively. Following staining the drivelines were rinsed in saline (0.9%) for 30 s, mounted onto coverslips using ProLong^®^ Diamond Antifade Mountant (Life Technologies, Australia) and immediately imaged on a Zeiss AxioVert A1 FL-LED microscope (Zeiss, Germany) utilizing a 100 × oil immersion objective. Images were analyzed using the instrument software (Zen 2.3).

### Statistical Methods

DEDs from 3 different donors were used for all experiments reported herein. In each experiment, DEDs from the same donor were tested in triplicate for each group (intact, PCL, un-seeded, pre-seeded). A total of 8 samples per group were analyzed by histology and a minimum of 6 for bacterial viability counts. CFU were enumerated by plating on agar plates and presented as CFU per ml of HSE homogenate. CFU from technical repeats were averaged and the average of each biological repeat is presented in dot plots per group. Group medians were compared for statistical differences by the non-parametric Kruskal Wallis test.

## Results

### Establishment of an *in vitro* Human Skin Equivalent Driveline Infection Model

**FIGURE 3:**
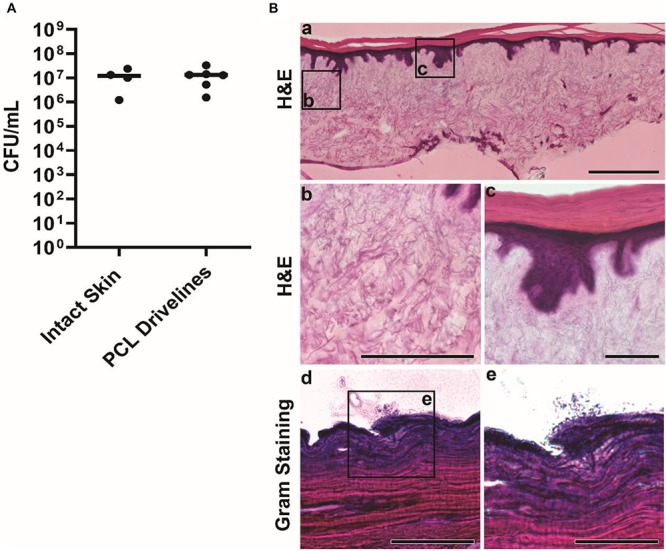
*Staphylococcus aureus* infection of intact HSEs. **(A)** Number of viable *S. aureus* colony forming units (CFU) recovered from intact skin HSEs (no driveline) or HSEs implanted with smooth PCL drivelines at 24 h post inoculation. Bacterial numbers are presented as CFU/mL with group means shown as horizontal lines. Dot plots show data from a minimum of 4 experimental repeats using HSEs derived from 3 skin donors. **(B)** Histological analysis of infected intact HSEs showing an overview of cross sections of the entire skin construct by H&E staining. Visible features include rete ridges between the epidermis and dermis (panel a, scale bar: 500 μm), connective dermal tissue (panel b, scale bar: 200 μm) and the epidermis and stratum corneum (panel c, scale bar: 100 μm). Gram staining of tissue cross-sections revealed *S. aureus* localizing exclusively on the uppermost layer of the epidermis in loose association with the skin tissue (panels d and e, scale bars: 100 μm and 50 μm, respectively).

*Staphylococcus aureus* is the leading cause of driveline infections (Koval and Stosor, 2019) and has been previously shown to remain confined to the outermost layer of the epidermis in an HSE wound infection model (Shepherd et al., 2009). This makes it an ideal organism for studying driveline-specific infections in HSEs *in vitro* as it does not invade into deeper tissue, unlike the Gram-negative wound pathogen *Pseudomonas aeruginosa* (Shepherd et al., 2009), ensuring that any bacterial penetration can only occur via the driveline opening. To confirm this in our model, we used reference *S. aureus* ATCC 29213 cultures to inoculate intact HSEs, devoid of a driveline, and HSEs with a PCL driveline mimicking the smooth surface of those currently used in clinical practice. *S. aureus* was recovered at 24 h post inoculation (hpi) in similar numbers from both HSE groups, with an average of 1.2 × 10^7^ CFU/mL recovered from intact HSEs and 1.3 × 10^7^ CFU/mL from HSEs implanted with the PCL driveline (Figure 3A). As the number of bacteria recovered at 24 hpi was similar to the number of inoculated bacteria, this suggests that *S. aureus* ATCC 29213 remained viable in our experimental model over this time-period maintaining a high tissue load.

**FIGURE 4:**
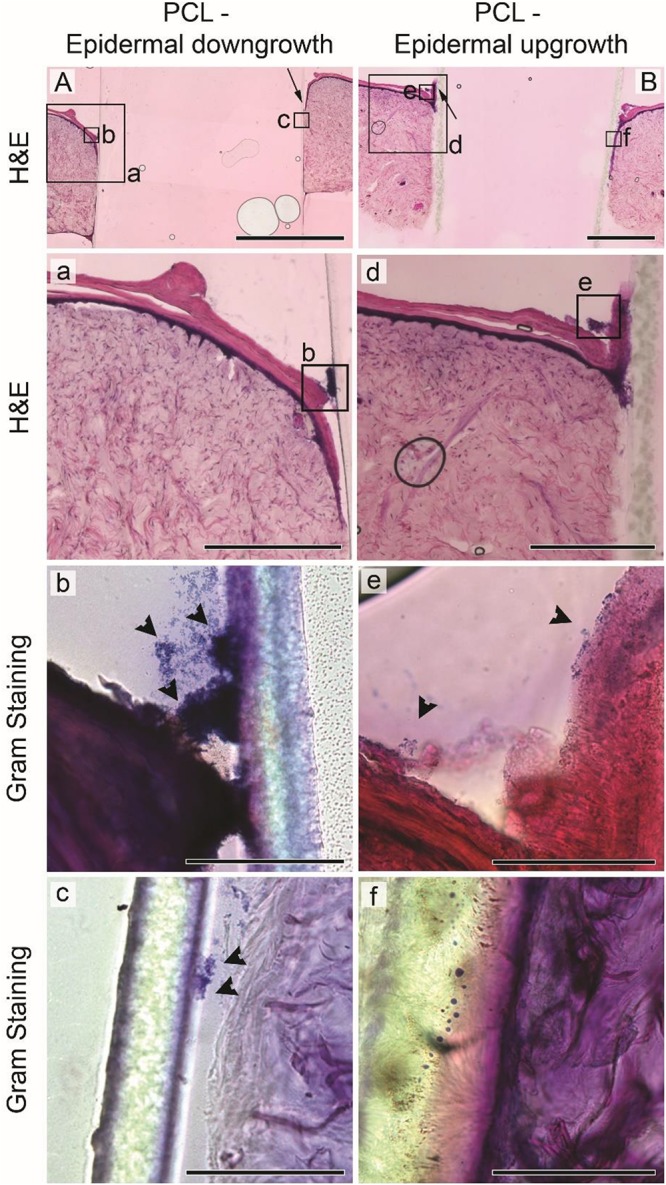
*Staphylococcus aureus* infection of HSEs implanted with smooth drivelines. Histological analysis by H&E staining of cross sections of HSEs implanted with a PCL mock driveline, exhibiting **(A)** epidermal downgrowth or **(B)** epidermal upgrowth, as indicated by the black arrows (scale bars: 2 mm). Panels (a) and (d) show digital magnifications of marked regions from H&E images shown in **(A,B)**, scale bars: 500 μm. Irrespective of epidermal growth pattern, Gram staining revealed bacterial colonization at the driveline entry point [panels (b) and (e)], denoted by black arrowheads, but limited or no presence of bacteria further along the skin – driveline interface [panels (c) and (f), scale bars 100 μm]. All images are from sections from the same samples with specific regions marked by black boxes in **(A,B)**.

While bacterial numbers recovered from tissues were similar, the localization of *S. aureus* differed between HSE groups (Figures 3B, 4). Gram staining of cross sections from intact HSEs revealed that *S. aureus* cells were exclusively present in the apical surface of the skin, with bacteria loosely associated with the uppermost layer and no bacteria present in deeper epidermal layers (Figure 3B). Similar analysis of HSEs implanted with PCL drivelines (Figure 4) revealed heavy *S. aureus* colonization at the driveline-skin exit site (inoculation site), with minimal bacterial presence observed further down this interface. Where bacteria were seen to be present deeper into the skin-driveline interface, they appeared to be mostly found in areas where gaps were present between HSE tissue and driveline (Figure 4).

H&E staining confirmed that all HSEs displayed a tissue morphology closely resembling that of native human skin (Figures 3B, 4). The epidermal growth patterns along the smooth PCL drivelines varied, with 5 out of 8 samples exhibiting epidermal downgrowth around the implant and 3 showing epidermal upgrowth. This is in line with our previous findings using smooth PCL drivelines in HSEs (Bolle et al., 2019) and suggests that the presence of *S. aureus* does not affect epidermal growth patterns.

Collectively, our microbiological and histological analyses suggest that the HSE model is able to sustain a high bacterial load without *S. aureus*-induced tissue damage or invasion of deeper epidermal and dermal layers. Bacterial non-invasiveness was considered a pre-requisite for an HSE-driveline infection model which sought to establish bacterial migration along the opening created by the presence of a driveline. Further, the results with the PCL drivelines suggest that the presence of the driveline does not affect total bacterial counts and that most bacteria are localized at the driveline exit site or associated with the driveline surface as a biofilm. We observed little *S. aureus* migration along the PCL implant interface and only where there were micro-gaps present, similar to recent clinical observations from explanted patient drivelines (Qu et al., 2019a). Thus, we conclude that our established *in vitro* HSE-driveline infection model is a suitable platform for investigating differences in the biological seal created by different driveline surfaces, which we investigated next.

### *S. aureus* Forms a Biofilm at the Entry Site of Porous Drivelines and Migrates Along the Driveline-Tissue Interface via Micro-Gaps

**FIGURE 5:**
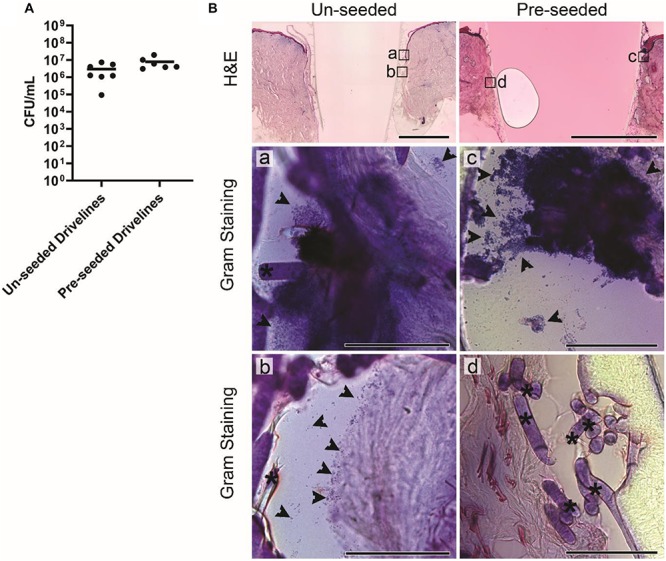
*Staphylococcus aureus* infection of HSEs implanted with porous drivelines. **(A)** Number of viable *S. aureus* recovered from HSEs with un-seeded or pre-seeded drivelines at 24 hpi. Bacterial numbers are presented as CFU/mL with group means shown as horizontal lines. Dot plots show data from a minimum of 6 experimental repeats using HSEs derived from 3 donors. **(B)** Histological analysis of infected HSEs implanted with un-seeded and pre-seeded drivelines retained an intact epidermis and dermis as shown by H&E staining of cross-sections (top panels; scale bars denote 2 mm). In HSEs with un-seeded drivelines, *S. aureus* (indicated by black arrows) localized at the inoculation site (a), with bacteria detected further along the interface and associated with deeper HSE tissue or present in gaps between the tissue and un-seeded driveline (b; Gram staining, left panel; scale bars denote 100 μm). In HSEs with pre-seeded drivelines, *S. aureus* localized at the inoculation site (c), but no bacterial colonization of the tissue in close contact with the driveline was observed (d; Gram staining, right panels; scale bars: 100 μm). Black asterisks denote PCL fiber struts.

To test whether skin integration around different porous driveline surfaces translates to differences in bacterial migration along the driveline, we inoculated HSEs with *S. aureus* as above, but this time the HSEs were implanted with MEW un-seeded and pre-seeded mock drivelines. The *S. aureus* tissue load recovered at 24 hpi was 3 × 10^6^ CFU/mL and 8 × 10^6^ CFU/mL, for the un-seeded and pre-seeded groups, respectively (Figure 5A). Histological staining revealed infected HSEs with un-seeded and pre-seeded drivelines to have an intact dermis and epidermis, with the lateral side of the pre-seeded drivelines exhibiting an intact thin layer of fibroblasts covering the scaffolds (Figure 5B– top right panel). All HSEs bearing un-seeded implants (8 out of 8) displayed epidermal downgrowth, but epidermal growth patterns around pre-seeded implants varied, with 4 exhibiting epidermal upgrowth and 4 epidermal downgrowth.

**FIGURE 6:**
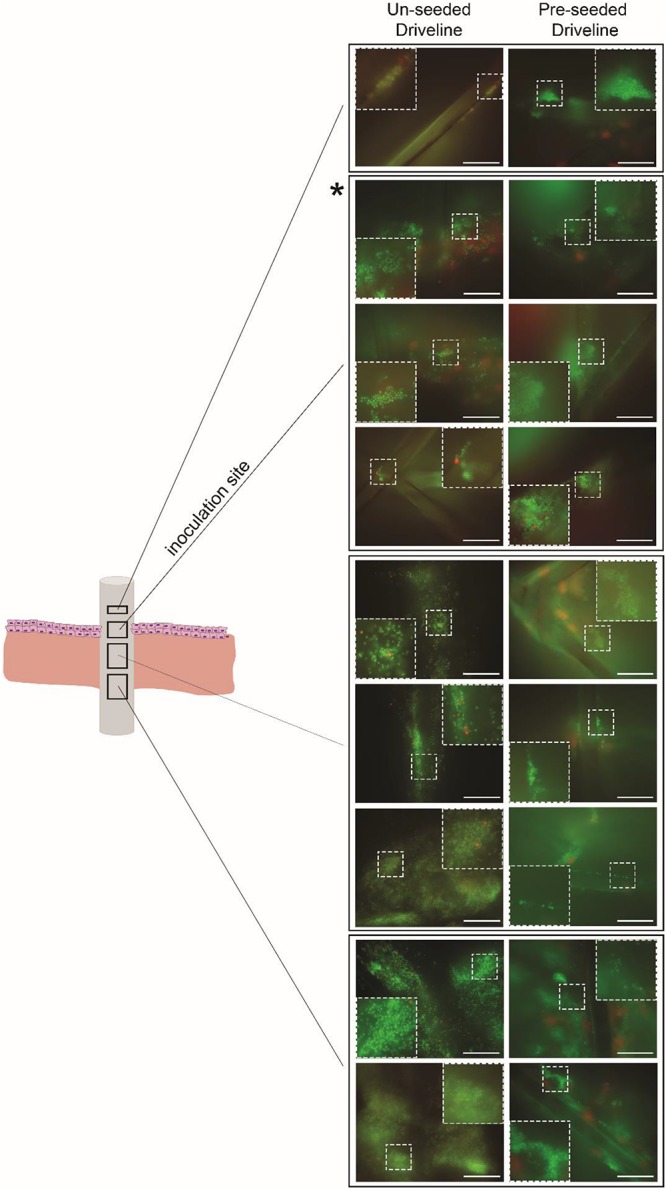
Fluorescence microscopy of infected HSE-explanted drivelines. *S. aureus* cells were stained with bacterial LIVE/DEAD staining (live = green, dead = red). Live *S. aureus* cells (seen as green cocci) were present in the voids and on the fiber struts of the un-seeded and pre-seeded drivelines at the inoculation site (second quadrant from top, denoted by asterisk) and deeper down the driveline surface (left and right panels, respectively; Scale bars: 30 μm). Inserts show a digitally zoomed section (25%) of the region marked by the white dotted line.

*Staphylococcus aureus* colonization of the driveline-skin entry site was invariantly observed in both HSE groups but was only noticeable further along the driveline-HSE interface where gaps were present between the driveline and the surrounding HSE tissue. Only small *S. aureus* clusters were observed to be associated with the deeper dermis interface, and only in HSEs implanted with un-seeded drivelines (Figure 5B), as these contained more gaps with the HSE tissue compared to pre-seeded or PCL drivelines. Similar to what was observed for PCL implants, *S. aureus* had formed a biofilm on the porous drivelines at the inoculation site, as revealed by LIVE/DEAD staining of explanted un-seeded and pre-seeded drivelines (Figure 6). Fluorescence microscopy along the entire length of the porous drivelines revealed *S. aureus* presence within the voids of the scaffold and along its fiber struts. *S. aureus* colonization and migration appeared to be more pronounced on the un-seeded drivelines, compared to pre-seeded PCL drivelines (Figure 6).

Taken together, our results suggest that epidermal growth pattern differences do not appear to strictly dictate whether *S. aureus* cells could migrate down the HSE-implant interface. Bacterial presence along the driveline surface was mostly associated with the presence of gaps along the tissue-driveline interface, which occurred more frequently in un-seeded porous drivelines. Moreover, challenging the skin-driveline interface with a high bacterial load did not impact the pre-established epidermal growth patterns at the implant entry site or the deeper dermal-implant interface, irrespective of the implant’s surface.

## Discussion

Infections around percutaneous implants and drivelines are a prevalent clinical problem, since the advent of the earliest VADs, limiting successful outcomes of ventricular assist device therapy (Hannan et al., 2019). It has been hypothesized by us and others that increased skin and tissue integration around the percutaneous device translates to a better biological seal against bacteria, with the potential to reduce bacterial infection rates (von Recum, 1984; Tagusari et al., 1998; Choi et al., 1999; Fukano et al., 2010; Isackson et al., 2011; Bolle et al., 2019). The aim of this study was thus to investigate whether porous scaffolds that were previously shown to increase skin integration and device stability in an HSE model would create a protective biological seal against pathogen entry. This aim necessitated the establishment of the first HSE-driveline infection model for *in vitro* investigations.

*In vitro* skin models have greatly advanced the field of cutaneous research and are based on excised full thickness skin, or tissue engineered skin with the dermal component based on synthetic matrices or de-cellularised dermis (Abd et al., 2016; Pupovac et al., 2018). The de-cellularised dermis skin model (DED-HSE) is well characterized, closely resembles native human skin and has been shown to re-epithelialise (Topping et al., 2006; Xie et al., 2010; Fernandez et al., 2014). More importantly, it has been used to study bacterial infections of wounds and the efficacy of functionalized biomaterials in reducing the bacterial burden in infected skin (Shepherd et al., 2009, 2011; Zheng et al., 2019).

*In vitro* skin models, however, have not yet been utilized to study bacterial infections around drivelines. Current knowledge on driveline infections is mainly derived from *in vitro* agar or drip flow bioreactor models, *ex vivo* analysis of explanted drivelines or *in vivo* rodent models (Arrecubieta et al., 2009; Toba et al., 2011; Qu et al., 2019b, a). The *in vitro* agar model was established by Qu et al. (2019b) to mimic microbial biofilm growth on drivelines within the subcutaneous tissue tunnel. The model consists of casting a Hinton or Roswell Park Memorial Institute medium agar mold by placing a piece of smooth driveline into molten agar and removing the driveline upon setting of the agar. Following insertion of an inoculated piece of smooth or porous driveline, the tunnel is covered with a lid from the same agar. To mimic the driveline exit site, the authors use a drip-flow biofilm reactor, whereby they place inoculated pieces of smooth or porous driveline into the incubation chamber while pumping growth medium through the system (Qu et al., 2019b). While these agar models offer suitable *in vitro* models to study biofilm formation and bacterial migration in the subcutaneous tissue, they do not allow examination of epidermal and dermal integration or its association with bacterial migration. *Ex vivo* clinical samples, on the other hand, are highly pertinent to studying various aspects of driveline infections, but observations tend to be subject to biological variation, demanding analyses of large numbers of clinical samples, and as such do not lend themselves as an ideal experimental platform to validate hypotheses. Small animal models have been utilized to study the *in vivo* role of bacterial virulence factors in driveline colonization and infection, such as the SdrF surface protein of *S. aureus* (Arrecubieta et al., 2009) and more generally the role of biofilm formation on driveline infections by *S. aureus* and *S. epidermidis* (Toba et al., 2011). None of these studies, however, directly investigated the role of skin integration and epidermal growth patterns in protecting from bacterial infection, so the clinical value of rodent driveline infection models to study skin integration remains unknown.

To address the lack of physiologically relevant *in vitro* models to study the correlation between skin integration and bacterial infection around percutaneous drivelines, we established a first-in-field *in vitro* driveline infection model based on the established DED-HSE model. Our model reproduces the clinical features observed in explanted drivelines from infected patients and provides a suitable tool for the investigation of skin-driveline integration properties on infection outcome. To the best of our knowledge, this is the first study to directly test whether increased skin integration around porous drivelines translates to an increased biological seal against pathogen entry.

Our study utilized *S. aureus* as the model pathogen, given *S. aureus* is the most common causative agent of driveline infections (Koval and Stosor, 2019). We deliberately inoculated the driveline exit-site with a relatively high bacterial burden (10^7^ CFU of *S. aureus* deposited in a small volume directly at the exit site), to challenge the biological seal created by the different driveline surfaces. Despite the high challenge, histological analysis confirmed that a high bacterial load could be maintained without tissue damage even up to 72 h post inoculation (data not shown). The non-invasiveness of *S. aureus* strains in HSE models, previously reported (Shepherd et al., 2009) and confirmed in this study for reference strain ATCC 29213, was important in ensuring that bacterial entry and migration occurred *via* the driveline and not *via* the tissue.

Our model also reproduced the formation of a sinus tract as a result of epidermal downgrowth around the implanted driveline. Sinus tract formation is a well-known mode of failure of percutaneous devices (von Recum, 1984) and has been hypothesized to create an environment conducive to bacterial proliferation, leading to subsequent device infection (Isackson et al., 2011; Affeld et al., 2012). Our study offers the first *in vitro* validation of this hypothesis, providing evidence of increased bacterial localization in the sinus tract between the driveline and the surrounding tissue. While in our model epidermal upgrowth was also observed, epidermal downgrowth was more frequently associated with implants that did not display tight integration with the surrounding HSE tissue and contained frequent micro-gaps along the length of the interface where bacteria were commonly observed. A recent analysis of infected drivelines explanted from patients undergoing heart transplants revealed insufficient integration between the Dacron^TM^ and the surrounding subcutaneous tissue with bacterial presence within similar voids (Qu et al., 2019a). In an *in vitro* study by the same group, challenge of porous Dacron^TM^ with the same *S. aureus* strain used in our study, also resulted in biofilm formation within the inter-fiber space of the intricate porous Dacron^TM^ structure (Qu et al., 2019b), similar to what we observed with the scaffold voids of mainly un-seeded and some pre-seeded drivelines. These results indicate that a healthy and well-integrated driveline-interface is crucial to reduce bacterial colonization and prevent migration along the implant interface. When the cell viability of the fibroblast layer forming the tight interface in our pre-seeded driveline-HSEs declined at 72 hpi, we observed increased bacterial migration into the driveline, further supporting this tenet (data not shown).

While there appears to be a consensus from *in vitro* and *ex vivo* studies on bacterial migration along porous drivelines, findings from animal models seem to be conflicting. A study by Isackson et al. (2011) in rabbits investigated four different types of titanium implants, all consisting of a percutaneous post and a subcutaneous anchor, with a smooth, porous or mixed surface (porous percutaneous post and smooth anchor or vice versa). Implants were repeatedly challenged with high-doses of *S. aureus* 4 weeks post implantation until manifestation of Grade II clinical signs of infection (experimental end point). Histological analysis revealed epidermal downgrowth and sinus tract formation in all implants, but implants with smooth components had a 7-fold increased risk of infection compared to implants with a porous surface in one or both components (Isackson et al., 2011). It is noteworthy that while the authors confirmed *S. aureus* to be the only organism isolated from infected implants, bacterial loads from implants or tissues were not determined and localization of bacteria along the implant interface was not examined either. Similarly, older studies in rabbits and pigs reported no signs of infection with porous carbon percutaneous devices (Krouskop et al., 1988; Nowicki et al., 1990). Differences in implant materials could account for these differences in results, as well as the use of different bacterial pathogens, which are known to have distinct biofilm formation properties.

Perhaps the most obvious difference, however, between *in vitro* and *in vivo* studies, is the absence of immune cells from most *in vitro* human skin models. Less than a handful of reconstructed HSE models containing immune cells have been reported to date (Bechetoille et al., 2007; Ouwehand et al., 2011; Linde et al., 2012; Kühbacher et al., 2017), which is testament to the complexity of establishing and maintaining 3D co-culture models (Pupovac et al., 2018). Overcoming those limitations in the near future will greatly enhance the clinical value of *in vitro* HSE-based models, including our HSE-driveline infection model, given the primary role of immune cells in fighting invading pathogens. In addition, the model could be further enhanced by adding movement of the driveline in the HSE through a bioreactor, in order to mimic driveline movement caused by patient breathing and other motion. While in this study we aimed to compare the biological seal created by different porous driveline surfaces (un-seeded and pre-seeded), which were previously shown to promote different skin integration patterns (Bolle et al., 2019), our model lends itself nicely for other types of studies, e.g., comparative efficacy of different driveline materials (Dacron, silicone, etc.) or testing of new and existing antibacterials and disinfectants. Furthermore, the model allows investigation of host-pathogen interactions and could be useful for *in vitro* studies of bacterial factors contributing to skin and/or driveline colonization and translocation. While, in our study we chose to explore bacterial localization in HSE tissues and drivelines by staining and microscopy -analyses which allow bacterial localization but generate image data not optimal for quantitative measurements-, quantification of viable bacteria in the tissue (as done in our study) could be combined with enumeration of bacteria on the HSE-explanted drivelines (as done in other studies; Qu et al., 2019b, a), to provide total numbers of bacteria colonizing the entire model system. Bacterial migration through the driveline could also be quantified by enumerating viable CFU recovered from media surrounding the HSE in each well (Figure 1), but we would highly recommend using transwells for such investigations to exclude bacteria “spill-over” from the epidermal inoculation site, especially for small-sized HSE constructs (Bolle and Totsika, unpublished observations).

Despite certain limitations, the *in vitro* percutaneous driveline infection model reported herein represents a versatile tool to further our understanding of factors leading to driveline infections and the role of skin integration and driveline surface in reducing bacterial infections. Such models can also provide a valuable early preclinical platform for the testing of new antimicrobial surfaces and therapeutics aimed at reducing the prevalence of device-related infections burdening health systems worldwide.

## Data Availability Statement

The datasets generated for this study are available on request to the corresponding author.

## Ethics Statement

The studies involving human participants were reviewed and approved by the Queensland University of Technology Research Ethics Committee (1300000063) and Uniting Healthcare/St Andrew’s Hospital Ethics Committee (0346). The patients/participants provided their written informed consent to participate in this study.

## Author Contributions

EB and MT conceived the study and designed the experiments, analyzed and interpreted the data and wrote the manuscript. EB, AV, and RD conducted the experiments. JF, TP, and TD contributed to the design and implementation of the research. All authors revised the manuscript for content and approved the final version.

## Conflict of Interest

The authors declare that the research was conducted in the absence of any commercial or financial relationships that could be construed as a potential conflict of interest.
